# Supplementary material for: MetaRibo-Seq measures translation in microbiomes
Source: Nat Commun. 2020 Jun 29;11:3268. doi: 10.1038/s41467-020-17081-z (PMC7324362; doi:10.1038/s41467-020-17081-z)
Supplement: Supplementary file 10 — Supplementary Data 7 [file 41467_2020_17081_MOESM10_ESM.zip › File2/Confidence_VeryHigh_Taxonomy/410481_out.krona.html]

Javascript must be enabled to view this page.

members
magnitude
magnitudeUnassigned
count
unassigned
taxon
rank

410481\_out

15

2
superkingdom
15

phylum
15
1239

186801
class
15

186802
15
order

216572
15
family

459786
genus
15


SRS014948\_contig\_number\_8134SRS018541\_contig\_number\_12049SRS054905\_contig\_number\_21176SRS055017\_contig\_number\_36147SRS105153\_contig\_number\_34409SRS143342\_contig\_number\_9926SRS143876\_contig\_number\_35556SRS143895\_contig\_number\_23814SRS143895\_contig\_number\_30675SRS146888\_contig\_number\_3155SRS147425\_contig\_number\_9378SRS148196\_contig\_number\_26921SRS148319\_contig\_number\_1621SRS148424\_contig\_number\_4264
1945593
14
species

1897012
1
species

SRS1055049\_contig\_number\_contig-100\_518.88206
